# Precision and Power: A Comprehensive Review of Exploring the Role of Laser Treatment in Hemorrhoidal Management

**DOI:** 10.7759/cureus.60011

**Published:** 2024-05-09

**Authors:** Dheeraj Surya, Pankaj Gharde

**Affiliations:** 1 General Surgery, Jawaharlal Nehru Medical College, Datta Meghe Institute of Higher Education & Research, Wardha, IND

**Keywords:** cost-effectiveness, pain management, clinical outcomes, minimally invasive, laser treatment, hemorrhoids

## Abstract

Hemorrhoids are a prevalent condition that significantly impacts the quality of life of affected individuals. Traditional treatment modalities range from conservative management to invasive surgical procedures, each with varying degrees of effectiveness and patient burden. Recently, laser treatment has emerged as a promising alternative, offering a minimally invasive approach with the potential for reduced complications and faster recovery. This comprehensive review aims to evaluate the role of laser treatment in hemorrhoidal management, exploring its mechanisms, clinical outcomes, safety, and economic implications. Through an extensive literature review and analysis of clinical trials, this paper assesses the efficacy of laser therapy compared to conventional treatments, highlighting its advantages in pain reduction, healing times, and patient satisfaction. The review also discusses the different types of lasers, including diode and Nd:YAG, and their specific applications in hemorrhoidal treatment. The findings indicate that laser treatment can be an effective and safe option for patients, encouraging its consideration as part of standard hemorrhoidal care protocols. However, gaps in long-term outcome data and the need for further studies on cost-effectiveness are identified. The review concludes with recommendations for future research, the advancement of laser technology, and the potential integration of laser treatment into clinical practice, aiming to enhance patient outcomes in hemorrhoidal management.

## Introduction and background

Hemorrhoids, also known as piles, are vascular structures in the anal canal that play a crucial role in fecal continence [1]. They can become swollen or inflamed due to various factors, leading to symptoms such as pain, itching, bleeding, and discomfort during bowel movements. Hemorrhoids are a common medical condition affecting a significant portion of the population worldwide. While often benign, they can significantly impact an individual’s quality of life, prompting the need for effective management strategies [2]. The management of hemorrhoids encompasses a spectrum of treatment modalities, ranging from conservative measures to surgical interventions. Conservative approaches typically involve lifestyle modifications, dietary changes, and over-the-counter medications to relieve symptoms [3]. Office-based procedures such as rubber band ligation (RBL) and sclerotherapy are commonly employed for mild to moderate cases, while surgical interventions like hemorrhoidectomy are reserved for severe or refractory cases. Despite the availability of these treatments, challenges still need to be addressed in terms of efficacy, invasiveness, and patient satisfaction [4].

In recent years, laser therapy has emerged as a promising alternative for the management of hemorrhoids. Laser treatment involves the application of focused light energy to target and coagulate hemorrhoidal tissue precisely, leading to shrinkage and resolution of symptoms [5]. This approach offers several potential advantages over traditional methods, including reduced postoperative pain, faster recovery times, and minimal risk of complications. However, the adoption of laser treatment in clinical practice varies, and its efficacy and safety profiles warrant a comprehensive evaluation [6]. The purpose of this review is to provide a comprehensive overview of the role of laser treatment in the management of hemorrhoids. By synthesizing existing literature, clinical evidence, and expert insights, we aim to elucidate the mechanisms of laser therapy, evaluate its clinical effectiveness and safety, and assess its potential impact on patient outcomes and healthcare economics. Furthermore, we seek to identify critical research gaps, future directions, and practical considerations for integrating laser treatment into clinical practice. Through this exploration, we aim to inform clinicians, researchers, and healthcare stakeholders about the evolving landscape of hemorrhoidal management and the role of laser technology therein.

## Review

Traditional treatment modalities for hemorrhoids

Conservative Management

Conservative management for hemorrhoids encompasses dietary and lifestyle modifications, medical treatments, and nonsurgical procedures. Dietary and lifestyle adjustments involve increasing fiber intake, staying hydrated, and avoiding straining and constipation [7,8]. Medical interventions include topical agents like steroids and hydrocortisone to alleviate symptoms of itching and inflammation and over-the-counter pain relievers such as acetaminophen, aspirin, and other non-steroidal anti-inflammatory drugs to manage pain and swelling [9]. Nonsurgical procedures, such as RBL, ablation, sclerosis, or necrosis of mucosal tissues, serve as the primary treatment for first- and second-degree internal hemorrhoids unresponsive to conservative measures [8]. These procedures aim to eliminate internal hemorrhoids and are typically administered by experienced clinicians with comparable efficacy [8]. Lord dilatation, involving manual stretching of the anal canal under anesthesia, is rarely practiced in the United States due to concerns about disrupting the sphincter mechanism [8]. Encouraging patients to retrain their toilet habits can also aid in reducing straining and constipation, leading to the shrinkage of internal hemorrhoids and the alleviation of symptoms [8]. Home treatment is often sufficient for most external hemorrhoids, involving gradually adding fiber to meals, increasing water intake, and using ointments to relieve itching [9]. Similarly, these measures can be employed for most internal hemorrhoids, although severe cases may necessitate medical intervention [9].

Office-Based Procedures

Office-based procedures for hemorrhoids offer minimally invasive options for treating grades I to III hemorrhoids that have not responded to conservative management [2]. These procedures include RBL, sclerotherapy, and infrared coagulation. RBL is the preferred choice due to its effectiveness [10]. During RBL, a ligation instrument is inserted through a speculum to grasp or suction the targeted hemorrhoid, and a rubber band is placed at its base, inducing ischemia and subsequent necrosis of the prolapsed mucosa. This results in the elevation of the inferior anal mucosa, providing relief. Although RBL is fast, easy to learn, and generally well tolerated, bleeding and pain are common complications. Sclerotherapy involves injecting a sclerosing agent into the hemorrhoidal tissue, promoting fibrosis and shrinkage of the hemorrhoid. While it is less effective than RBL in symptom control, it is associated with reduced post-procedural pain [4]. Infrared coagulation utilizes infrared light to induce thermal injury to the hemorrhoidal tissue, leading to necrosis and fibrosis. Although less effective than RBL, it tends to cause more post-procedural pain [4]. Other office-based procedures, such as cryotherapy, radiofrequency ablation, and laser therapy, are less commonly employed. Their efficacy and safety profiles are less well established than those of RBL, sclerotherapy, and infrared coagulation [2]. Generally, office-based procedures offer advantages such as reduced postoperative pain, shorter recovery times, and lower complication rates compared to surgical interventions. However, recurrence rates can be significant, necessitating additional treatments [2,10]. Therefore, close monitoring for complications such as fever and urinary problems is essential, and appropriate management of pain and bleeding is crucial [10].

Surgical Interventions

Surgical interventions for hemorrhoids encompass procedures such as hemorrhoidectomy, transanal hemorrhoidal dearterialization (THD), and the procedure for prolapse and hemorrhoids (PPH). Excisional techniques, like hemorrhoidectomy, have demonstrated comparable outcomes regarding pain levels, time to resume normal activities, and complication rates [11]. However, they are linked to a higher recurrence risk than nonexcisional methods such as THD and PPH [11]. THD and PPH are associated with reduced postoperative pain and lower complication rates but carry higher recurrence rates post-surgery [11]. THD involves ligating the hemorrhoidal arteries transanally, while PPH employs a circular stapler to excise hemorrhoidal tissue and reposition the remaining tissue into the anal canal [11]. A systematic review and meta-analysis encompassing 98 trials and 7,827 patients, evaluating 11 surgical treatments for grades III and IV hemorrhoids, concluded that stapled hemorrhoidopexy and THD were correlated with decreased postoperative pain but exhibited higher recurrence rates compared to other surgical interventions [8]. The selection of the appropriate surgical intervention hinges on several factors, including the severity of the hemorrhoids, patient preferences, and the surgeon’s proficiency [11]. It is imperative to weigh each surgical procedure’s potential benefits and risks and thoroughly discuss it with the patient before deciding. In addition to surgical interventions, alternative treatment options for hemorrhoids encompass RBL, infrared coagulation, sclerotherapy, anal dilatation, bipolar coagulation, and adherence to a high-fiber diet [12]. The selection of the treatment modality is contingent upon factors such as the severity of the hemorrhoids, patient preferences, and the physician’s expertise.

Effectiveness and Limitations

Laser treatment for hemorrhoids has emerged as a promising option, demonstrating efficacy in reducing postoperative pain and morbidity when compared to conventional treatment methods. A study involving 21 patients with hemorrhoidal disease who underwent laser hemorrhoidoplasty (LHP) revealed encouraging outcomes, including the absence of symptom recurrence, minimal postoperative pain, and a low incidence of stenosis or incontinence [13]. LHP employs a diode laser generator set between 12 and 15 W in pulsed mode, delivering laser energy via a radial fiber [13]. This minimally invasive procedure has shown particular effectiveness in managing grades II and III hemorrhoids without significant prolapse [13], offering patients relief with minimal discomfort. Similarly, a study involving 51 patients with grade I, II, and III hemorrhoids treated with laser sclerotherapy and hemorrhoidal artery ligation (HAL) demonstrated favorable results without significant complications or mortality [14]. Conducted under epidural anesthesia, this procedure involves introducing a conical tip fiber of 1470 nm laser into the submucosal plane of each hemorrhoid and delivering energy up to 250 J per pile mass [14]. These findings underscore the safety and efficacy of laser-based interventions in managing hemorrhoids, providing patients with a potentially less invasive treatment option. Furthermore, a network meta-analysis of randomized controlled trials comparing various surgical interventions for grade III and IV internal hemorrhoids revealed that stapled hemorrhoidopexy is associated with a higher long-term recurrence rate compared to other procedures such as the PPH and Milligan-Morgan hemorrhoidectomy [15]. While stapled hemorrhoidopexy may offer particular advantages, such as reduced postoperative pain, its long-term efficacy should be carefully considered when determining the most appropriate treatment approach.

Laser treatment for hemorrhoids: mechanisms and techniques

Principles of Laser Therapy

**Figure 1:**
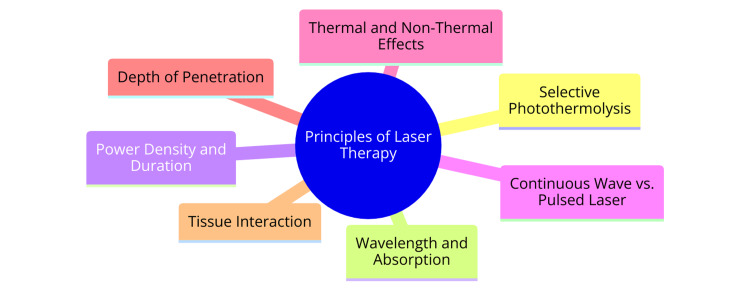
Principles of laser therapy Image credit: Dheeraj Surya

Laser treatment for hemorrhoids has emerged as a promising alternative, demonstrating efficacy in reducing postoperative pain and morbidity compared to conventional treatment methods. A study involving 21 patients with hemorrhoidal disease who underwent LHP yielded encouraging outcomes, including the absence of symptom recurrence, minimal postoperative pain, and a low incidence of stenosis or incontinence [13]. LHP utilizes a diode laser generator set between 12 and 15 W in pulsed mode, delivering laser energy via a radial fiber [13]. This minimally invasive procedure has shown particular effectiveness in managing grades II and III hemorrhoids without significant prolapse [13], providing patients with relief while minimizing discomfort. Similarly, a study involving 51 patients with grade I, II, and III hemorrhoids treated with laser sclerotherapy and HAL demonstrated favorable results without significant complications or mortality [14]. Performed under epidural anesthesia, this procedure involves introducing a conical tip fiber of 1470 nm laser into the submucosal plane of each hemorrhoid and delivering energy up to 250 J per pile mass [14]. These findings underscore the safety and efficacy of laser-based interventions in managing hemorrhoids, offering patients a potentially less invasive treatment option. Furthermore, a network meta-analysis of randomized controlled trials comparing various surgical interventions for grade III and IV internal hemorrhoids revealed that stapled hemorrhoidopexy is associated with a higher long-term recurrence rate compared to other procedures such as the PPH and Milligan-Morgan hemorrhoidectomy [15-17]. While stapled hemorrhoidopexy may offer particular advantages, such as reduced postoperative pain, its long-term efficacy should be carefully considered when determining the most appropriate treatment approach. The principles of laser therapy are shown in Figure 1.

Types of Lasers Used

Several methods of laser treatment for hemorrhoids exist, each offering minimally invasive options for effective management. Firstly, endolaser ablation involves delivering laser energy directly into the hemorrhoidal mass, causing it to collapse [18]. Secondly, the surface laser application employs laser energy directed onto the surface of the hemorrhoidal mass [18]. Total hemorrhoidoplasty with laser is a gentle intervention utilizing a specialized laser procedure lasting only 15 minutes and deemed painless [19]. Lastly, total laser hemorrhoidectomy, also known as total LHP, is an advanced laser application treating the entire length of the anal canal, shrinking the hemorrhoidal plexus comprehensively, and addressing all types of hemorrhoidal disease [19]. These various laser treatments provide patients with practical alternatives for managing hemorrhoids while minimizing invasiveness.

Surgical Techniques and Procedures

Hemorrhoid surgery aims to address swollen veins around the anus, which can lead to bleeding, pain, or discomfort. Standard surgical options include open or closed hemorrhoidectomy, stapled hemorrhoidopexy, and Doppler-guided HAL. Open or closed hemorrhoidectomy entails removing the hemorrhoidal sac, with the open approach leaving the mucosal defect edges unrepaired while the closed approach reapproximates them [20,21]. Typically reserved for prolapsing diseases, this procedure boasts a significantly lower recurrence rate than other methods [20]. Stapled hemorrhoidopexy involves excising excess tissue and repositioning prolapsing tissue with a unique tool, which is then secured [20]. Although associated with lower pain scores than excisional hemorrhoidectomy, it does not surpass it in terms of recurrence rates [20]. Doppler-guided HAL locates and ligates individual hemorrhoidal arteries using a Doppler probe, diminishing blood flow to the hemorrhoidal plexus and inducing tissue shrinkage and degeneration [20]. This procedure is linked to minimal postoperative pain and a low risk of stenosis or incontinence [20]. RBL is a common form of hemorrhoid surgery involving placing a rubber band around a hemorrhoid’s base, constricting its blood supply until it eventually detaches [21]. Often conducted in a doctor’s office, it may necessitate multiple procedures to remove a hemorrhoid [21] altogether. Coagulation therapy, another surgical approach, utilizes infrared light, heat, or extreme cold to prompt hemorrhoid retraction and shrinkage [22]. Typically performed alongside anoscopy at the doctor’s office, this method is minimally invasive. HAL identifies hemorrhoid-causing blood vessels via ultrasound and ligates or closes them off [22]. While potentially as effective as traditional hemorrhoidectomy, it employs less invasive techniques.

Advantages and Disadvantages

The advantages and disadvantages of laser treatment for hemorrhoids are shown in Table 1.

**Table 1:** Advantages and disadvantages of laser treatment for hemorrhoids

Advantages	Disadvantages
Precise tissue targeting: Laser treatment allows for precise targeting of hemorrhoidal tissue, minimizing damage to surrounding healthy tissue. This precision can result in more effective treatment outcomes and a reduced risk of complications [13].	Costlier compared to some traditional methods: Laser hemorrhoid treatment may be more expensive than traditional treatment options, which could limit access for some patients, particularly in healthcare systems with limited resources [11].
Minimal postoperative pain and discomfort: Patients undergoing laser treatment for hemorrhoids often experience less postoperative pain and discomfort compared to traditional surgical procedures. This can lead to a faster recovery and improved patient satisfaction [14].	Requires specialized equipment and training: Performing laser treatment for hemorrhoids requires specialized equipment and training, which may only be available in some healthcare settings. This could limit the widespread adoption of this treatment modality [14].
Reduced risk of postoperative bleeding: Laser treatment can effectively coagulate blood vessels, reducing the risk of postoperative bleeding. This can result in a smoother recovery process and lower rates of complications [15].	Limited availability in some healthcare settings: Laser hemorrhoid treatment may not be readily available in all healthcare settings, particularly in rural or underserved areas. This could restrict access to this treatment option for specific patient populations [13].
Faster recovery times: The minimally invasive nature of laser treatment typically leads to faster recovery times than traditional surgical procedures. Patients may be able to return to their normal activities sooner, improving their overall quality of life [16].	Variable efficacy based on hemorrhoid severity: The efficacy of laser treatment for hemorrhoids may vary depending on the severity of the condition. In some cases, more advanced or severe hemorrhoids may not respond as well to laser therapy, necessitating alternative treatment approaches [18].
Minimal risk of sphincter injury: Laser hemorrhoid treatment is associated with a lower risk of sphincter injury than traditional surgical techniques. This can help preserve anal function and reduce the risk of long-term complications, such as fecal incontinence [13].	Long-term data on outcomes: While laser treatment for hemorrhoids has shown promising short-term outcomes, more long-term data on its efficacy and durability should be needed. Further research is needed to assess this treatment modality’s long-term outcomes and recurrence rates [21].
Outpatient procedure: Laser treatment for hemorrhoids is often performed on an outpatient basis, meaning patients can undergo the procedure and return home the same day. This reduces the need for hospitalization and can lead to cost savings for patients and healthcare systems [10].	Potential for intraoperative complications: Like any medical procedure, laser treatment for hemorrhoids carries a risk of intraoperative complications, such as bleeding, perforation, or damage to surrounding tissues. Experienced healthcare providers should carefully consider and manage these risks [11].
Minimal risk of wound infection: Laser treatment does not involve incisions or open wounds, and wound infection is reduced compared to traditional surgical procedures. This can lead to improved patient outcomes and lower rates of postoperative complications [12].	Potential for recurrence: While laser treatment can effectively shrink hemorrhoidal tissue and alleviate symptoms in many cases, there is a risk of recurrence over time. Patients should be informed about the potential for recurrence and monitored closely for signs of symptom recurrence or disease progression [16].

Clinical evidence and outcomes

Review of Clinical Studies and Trials

LHP has garnered significant attention in clinical research, with multiple studies and trials showcasing promising results. In a preliminary study involving 21 patients undergoing LHP for hemorrhoidal disease, the procedure was performed under spinal or sling anesthesia without prior bowel preparation or colonic evacuation [5]. In pulsed mode, a diode laser generator set between 12 and 15 W delivered laser energy through a radial fiber (Leonardo 1470 from Biolitec). A windowed proctoscope and ice cubes were employed, with mucopexy conducted in cases of significant mucosal prolapse, followed by intermittent laser application. Postoperative care included analgesics, non-steroidal anti-inflammatories, metronidazole, laxatives, and antiseptic applications, with follow-up conducted weekly until healing and then every two months. The parameters studied encompassed demographics, medical history, symptoms, hemorrhoidal grade, intraoperative details, and short- to medium-term outcomes [5]. A randomized clinical trial comparing LHP to Milligan and Morgan hemorrhoidectomy demonstrated that LHP induces less postoperative pain over a shorter period and effectively resolves hemorrhoidal symptoms with lower morbidity [23]. Additionally, a double-blind controlled trial comparing LHP, excisional hemorrhoidectomy, and recto-anal repair found LHP to be a safe and effective treatment for symptomatic hemorrhoids, resulting in less postoperative pain and an earlier return to work compared to excisional hemorrhoidectomy [23]. Moreover, a pilot Australian study investigating LHP in the treatment of symptomatic hemorrhoids concluded that it represents a safe and effective treatment option, yielding minimal postoperative pain and favorable short- and long-term outcomes. It demonstrated superior short-term clinical outcomes compared to conventional hemorrhoidectomy (CH), with reduced morbidity and pain and earlier resumption of work or daily activities [24].

Comparative Effectiveness with Traditional Treatments

LHP has emerged as a more effective treatment option compared to traditional surgical methods, such as open surgical hemorrhoidectomy, for managing hemorrhoidal disease. A systematic review and meta-analysis of both randomized and nonrandomized studies revealed that LHP provides superior short-term clinical outcomes compared to CH in patients with grade II or III hemorrhoids [25]. LHP was associated with reduced morbidity and pain, as well as an earlier return to work or daily activities. Moreover, LHP demonstrated shorter operative time and less intraoperative blood loss than CH [25]. Postoperative pain was also significantly lower in the LHP group, with reduced use of analgesia and a quicker return to normal activities. In a study comparing LHP with open surgical hemorrhoidectomy in patients with third- and fourth-degree hemorrhoids, LHP exhibited superiority in terms of operative time and early postoperative pain [26]. The procedure time for LHP was notably shorter than for open surgery, and there was a statistically significant difference in early postoperative pain between the two groups [26]. Another study comparing LHP with conventional open surgical hemorrhoidectomy found that LHP was associated with shorter operative time, less postoperative pain, shorter hospital stays, and fewer postoperative complications [27].

Complication Rates and Long-Term Outcomes

The complication rates and long-term outcomes of LHP for grades II and III hemorrhoidal disease have been extensively studied in multiple clinical trials. A systematic review and meta-analysis revealed that LHP provides superior short-term clinical outcomes compared to CH, demonstrating reduced morbidity and pain and an earlier return to work or daily activities [25]. In a study involving 50 patients who underwent LHP for grades II and III hemorrhoidal disease, it was reported that 36 out of 50 patients (72%) experienced complete or significant symptom improvement at 60 days post-procedure [28]. Postoperative complications were observed in nine out of 50 patients (18%), with three cases classified as Clavien-Dindo grade IIIb complications (including two fistulas and one case of incontinence) [28]. However, complications after laser procedures were infrequent and encompassed postoperative bleeding, infection, external thrombosed piles, fistulas, and fissures [28]. In another study involving 100 patients with grades II and III hemorrhoids and minimal to mild rectal prolapse treated with the hemorrhoidal laser procedure (HeLP), it was found to be an effective, safe, and nonpainful procedure for managing patients with symptomatic second- or third-degree hemorrhoids with mild to minimal rectal mucosal prolapse [25]. Overall, LHP emerges as a safe and effective treatment option for hemorrhoidal disease, characterized by minimal postoperative pain and favorable short- and long-term outcomes. The complication rates are low, with infrequent occurrences such as postoperative bleeding, infection, external thrombosed piles, fistulas, fissures, and incontinence. The evidence suggests that LHP offers superior short-term clinical outcomes compared to CH, highlighting its potential as a preferred treatment option for grades II and III hemorrhoidal disease.

Patient Satisfaction and Quality of Life

The impact of laser treatment on patient satisfaction and quality of life in managing hemorrhoidal disease has garnered significant attention in research. Studies have indicated that LHP offers superior short-term clinical outcomes compared to CH, characterized by reduced morbidity, decreased postoperative pain, and earlier resumption of work or daily activities [25]. Research has delved into the effect of surgery on the primary symptoms of hemorrhoids and the patient’s overall quality of life, revealing that LH results in enhanced postoperative pain management, faster recovery, and heightened patient satisfaction in contrast to traditional surgical methods [29]. Furthermore, laser therapy is minimally invasive, resulting in less postoperative pain and minimal morbidity, factors that can positively impact patient satisfaction and quality of life during the recovery phase [5].

Future directions and emerging trends

Innovations in Laser Technology

Chirped pulse amplification (CPA) is a technique utilized to generate high-intensity laser pulses without causing damage to the material. It involves stretching optical bursts in time to decrease peak power, amplifying them, and then compressing them back into a high-intensity pulse. CPA is widespread in applications like corrective eye surgery [30]. Holographic data storage is an area of research exploring the utilization of holographic technology to enhance the data storage capacity of optical devices significantly. By storing data in three-dimensional holograms, the storage space can be substantially expanded compared to current surface-based optical storage methods [30]. Laser cooling, contrary to intuition, involves using lasers to cool substances by utilizing the momentum of photons to decelerate the movement of atoms and molecules. This laser cooling technique has diverse applications, including quantum computing and the detection of gravitational waves [30]. The development of high-power laser systems has led to the creation of compelling laser systems such as the Extreme Light Infrastructure Beamlines laser in the Czech Republic, capable of producing pulses of up to 10 petawatts (10 quadrillion watts). These powerful lasers find applications in advanced physics research, materials science, and medical fields [31]. Advancements in medical laser technology have resulted in the evolution of various types of lasers, including carbon dioxide, argon, excimer, Nd:YAG, and diode lasers, enabling a wide range of applications in surgery, ophthalmology, dermatology, dentistry, and oncology. Continuous technological innovations are expanding the potential of lasers in healthcare [31,32].

Potential Applications and Advancements

Effective pain alleviation: Carbon dioxide laser therapy, for instance, has demonstrated effective pain alleviation from the first session, providing patients with a more comfortable postoperative course [33].

Minimally invasive techniques: Advancements in laser technology have led to the development of minimally invasive procedures like HeLP. This technique is safe, effective, and nonpainful for managing symptomatic second- or third-degree hemorrhoids with mild rectal prolapse [33].

Reduced postoperative pain: Laser therapies, including intra-hemorrhoidal laser therapy, have been linked to less postoperative pain and shorter recovery times compared to traditional surgical methods such as hemorrhoidectomy [34].

Improved outcomes: Laser treatments have shown promising outcomes by reducing hemorrhoid symptoms, diminishing hemorrhoid size, and decreasing the frequency of pain, bleeding, and other symptoms associated with hemorrhoidal disease [34].

Cost-effectiveness: Studies have indicated that laser procedures like Doppler-guided hemorrhoidal laser therapy can be cost-effective, with lower total patient costs than other treatment modalities [34].

Ambulatory treatment option: Laser therapies, such as HeLP, have been recognized as suitable ambulatory treatments for patients with symptomatic second- or third-degree hemorrhoids. They offer a safe and effective alternative to traditional surgical approaches [34].

Research Gaps and Areas for Further Investigation

Long-term follow-up studies: While numerous studies have highlighted the positive outcomes of laser procedures for HD in the short to medium term, there is a pressing need for additional long-term follow-up studies. These studies would evaluate the durability of symptom resolution, recurrence rates, and potential late complications associated with laser treatments [35-37].

Comparative studies: More comparative studies are necessary to directly contrast the efficacy, safety, and long-term outcomes of laser procedures like LHP and HeLP with traditional surgical methods such as hemorrhoidectomy or RBL. These investigations could offer valuable insights into the optimal treatment approach for different grades of HD [35-37].

Patient-reported outcomes: Future research could focus on integrating patient-reported outcome measures to evaluate factors like quality of life, treatment satisfaction, and the impact on daily activities following laser procedures for HD. Understanding patient perspectives can aid in tailoring treatments to individual needs and enhancing overall patient care [35-37].

Optimal patient selection: Investigating the criteria for selecting patients who would benefit most from laser treatments, including factors such as the severity of hemorrhoids, rectal prolapse, and other individual characteristics, could refine treatment guidelines and enhance patient outcomes [35-37].

Technological advancements: Research efforts toward advancements in laser technology, such as Doppler-guided procedures and selective closure of hemorrhoidal arteries, could further refine the precision, efficacy, and safety of laser treatments for HD. Exploring novel techniques and tools may improve outcomes and decrease complications [36,37].

## Conclusions

In this comprehensive review, we explored the role of laser treatment in managing hemorrhoids, highlighting its efficacy and minimal invasiveness compared to traditional methods. Our findings reveal that laser therapy significantly reduces pain, expedites recovery, and minimizes complications, enhancing patient satisfaction and quality of life. As such, it emerges as a viable alternative for patients seeking less invasive treatments. The review suggests updating clinical guidelines to include laser treatment as a standard option and emphasizes the need for further training in laser technologies for healthcare providers. However, despite promising results, there remains a need for more extensive, randomized controlled trials to validate long-term outcomes and comparative effectiveness. Future research should also assess the cost-effectiveness of laser treatments in various healthcare settings to better understand their economic impact. Innovations in laser technology that increase precision and reduce recovery times could further solidify its position in hemorrhoidal treatment protocols. The potential of laser treatment aligns with broader trends toward precision and patient-centered medical treatment approaches, promising to significantly transform hemorrhoidal management. This review aims to inspire ongoing discussion and innovation in the field, paving the way for enhanced patient outcomes and a broader acceptance of new surgical technologies.
